# Photoperiod-Dependent Effects on Blood Biochemical Markers of Phenolic-Enriched Fruit Extracts

**DOI:** 10.1021/acs.jafc.4c01698

**Published:** 2024-05-29

**Authors:** Francesca Manocchio, Diego Morales, Elia Navarro-Masip, Gerard Aragonès, Cristina Torres-Fuentes, Francisca Isabel Bravo, Begoña Muguerza

**Affiliations:** † Nutrigenomics Research Group, Departament de Bioquímica i Biotecnologia, Universitat Rovira i Virgili, C/Marcel·lí Domingo s/n, 43007 Tarragona, Spain; ‡ Nutrigenomics Research Group, Institut d’Investigació Sanitària Pere Virgili, C/Marcel·lí Domingo s/n, 43007 Tarragona, Spain; § Center of Environmental, Food and Toxicological Technology (TecnATox), University Rovira i Virgili, C/Marcel·lí Domingo s/n, 43007 Tarragona, Spain

**Keywords:** glucose profile, insulin, lipid profile, photoperiods, (poly)phenols

## Abstract

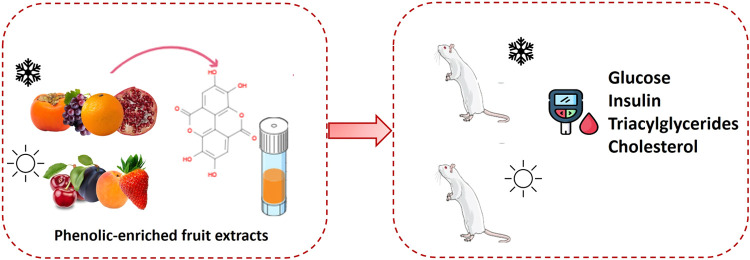

Fruits are rich in bioactive compounds, such as (poly)phenols, and their intake is associated with health benefits, although recent animal studies have suggested that the photoperiod of consumption influences their properties. Fruit loss and waste are critical issues that can be reduced by obtaining functional fruit extracts. Therefore, the aim of this study was to obtain phenolic-enriched extracts from eight seasonal fruits that can modulate blood biochemical parameters and to investigate whether their effects depend on the photoperiod of consumption. Eight ethanol-based extracts were obtained and characterized, and their effects were studied in F344 rats exposed to short (6 h light, L6) and long (18 h light) photoperiods. Cherry and apricot extracts decreased blood triacylglyceride levels only when consumed under the L6 photoperiod. Pomegranate, grape, and orange extracts reduced cholesterol and fasting glucose levels during the L6 photoperiod; however, plum extract reduced fasting glucose levels only during the L18 photoperiod. The results showed the importance of photoperiod consumption in the effectiveness of phenolic-enriched fruit extracts and promising evidence regarding the use of some of the developed fruit extracts as potential functional ingredients for the management of several blood biomarkers.

## 1 Introduction

The consumption of diets rich in fruits and vegetables has been linked to health benefits, such as the reduction of high blood pressure, blood triacylglyceride (TAG), and cholesterol levels.^1^ Nevertheless, between 4 and 7% in the United States and Europe and more than 10% in other countries, such as Africa and Latin America, fruit production is lost.^2^ Moreover, these losses have been found to be higher on farms, reaching values up to 6 times higher than off-farm losses.^3^ Although there are strategies to reduce these losses, including prolonging the half-life of these fruits during harvesting, storage, or transportation^4,5^ and to increase the consumption of more seasonal foods associated with health benefits,^6^ more strategies are needed. Thus, these unused fruits could be utilized to obtain extracts rich in bioactive compounds, which would allow their valorization into value-added products and contribute to the farm circular economy.

Although fruits are rich in many bioactive molecules, (poly)phenols are one of the main compounds responsible for their beneficial effects.^7^ Phenolic compounds are secondary plant metabolites that are produced in response to stress factors.^8^ They comprise a wide range of structures, with more than 8000 different compounds that contain at least one aromatic ring containing one or more hydroxyl groups.^9^ Flavonoids are the major phenolic compounds consumed (60%) and are subdivided into anthocyanidins, flavanols, flavanones, flavones, flavonols, and isoflavones.^10^ Several studies have reported that (poly)phenols can exert a wide range of effects, including reduction of dyslipidemia and insulin resistance.^11^ In particular, phenolic-rich extracts obtained from fruits such as pomegranate or grape have been demonstrated to be effective as antioxidant, antihyperglycemic, anti-hyperlipidemic, and cardioprotective agents, and many studies have been conducted not only in animal models but also in humans through clinical trials.^12,13^ Nevertheless, phenolic-induced health effects vary depending on the specific (poly)phenols consumed, dose, bioaccessibility, or bioavailability.^14^ In this regard, the profile and quantity of phenolic compounds in a specific fruit or vegetable are affected by plant type and variety, as well as by environmental, harvesting, storage, and transport conditions.^15^ In fact, sweet cherries from two different geographical origins showed different phenolic compositions and exerted different effects on liver and serum antioxidant markers in Fisher 344 (F344) rats after their consumption.^16^

In this context, the xenohormesis theory postulates that phenolic compounds can act as signal molecules in animals, providing information on external conditions and allowing them to adapt to environmental changes through variations in physiological, behavioral, and metabolic processes.^17^ Additionally, it has been suggested that (poly)phenols can help the adaptation of physiology to seasonal rhythms.^18,19^ Recent studies carried out in our research group have shown that the beneficial effects of fruits vary depending on the photoperiod during which they are consumed.^16,18^ For instance, the intake of sweet cherries increased serum insulin levels and the HOMA index only in F344 rats exposed to a short photoperiod (6 h light, L6).^20^ In contrast, animals exposed to a long photoperiod (18 h of light, L18) showed decreased and increased serum levels of nonesterified fatty acids and high-density lipoprotein cholesterol, respectively, compared to the levels observed in the control group exposed to the same photoperiod. However, no effects were observed when the same cherries were consumed by animals exposed to L6 and an intermediate photoperiod (12 h light, L12).^20^ Other studies have shown that the effects of a grape seed phenolic-enriched extract on the liver and adipose tissue depended on the photoperiod at which they were consumed.^21,22^ Considering these previous studies, the aim of this research was to obtain phenolic-enriched extracts from different seasonal fruits that are widely consumed in plant-based diets and evaluate their effects on blood biochemical parameters related to glucose and lipid metabolism in F344 rats exposed to different photoperiods.

## 2 Materials and Methods

### 2.1 Reagents

Analytical grade ethanol and formic acid were obtained from PanReac Química SLU (Castellar del Vallès, Barcelona, Spain) and Scharlab (Sentmenat, Barcelona, Spain), respectively. For the analysis of the basic composition of the samples, heat-stable α-amylase, protease from *Bacillus licheniformis*, and amyloglucosidase from *Aspergillus niger* were purchased from Sigma-Aldrich (Madrid, Spain). Folin-Ciocalteu reagent, gallic acid, and catechin were obtained from Sigma-Aldrich. Cyanidin-3-*O*-rutinoside was purchased from CyPhytoLab (Vestenbergsgreuth, Germany). All other reagents used were of analytical grade. For high-performance liquid chromatography (HPLC) analysis, *p*-coumaric acid (≥98%), quercetin (≥95%), ellagic acid (≥95%), pelargonidin (≥90%), catechin (≥97%), castalagin (≥95%), apigenin (≥95%), hesperidin (≥80%), and naringenin (≥95%) and other HPLC-grade reagents (formic acid, acetonitrile, acetic acid, dimethyl sulfoxide and methanol) were provided by Sigma-Aldrich.

### 2.2 Obtaining of Fruit Extracts

Ripe summer (cherry (*Prunus avium* L. var. *lapins*; Sierra el Frasno, Zaragoza, Spain), apricot (*Prunus armeniaca* L.), strawberry (*Fragaria vesca* L. var. *cadonga*; Reus, Tarragona, Spain), plum (*Prunus domestica* L. var. *black diamond*; Ruidoms, Tarragona, Spain)), and winter fruits (grape (*Vitis vinifera* L. var. *cabernet*; Constantí, Tarragona, Spain), orange (*Citrus sinensis* L. var. *navelina*; Cambrils, Tarragona, Spain), persimmon kaki (*Diospyros kaki* L.f. var. *rojo brillante*; Ruidoms, Tarragona, Spain), and pomegranate (*Punica granatum* L. var. *tendral de valencia*; Benissanet, Tarragona, Spain)) were acquired from local producers during the summer and winter seasons, respectively. Pedicels were manually removed, and whole fruits, including the skin, seeds, and pulp (since these components are normally consumed and contain relevant phenolic compounds), were mechanically triturated and mixed to obtain homogenates of strawberries and grapes. For cherry, apricot, plum, and persimmon, the seeds were removed before trituration, and for orange and pomegranate, the skins were removed before trituration. The homogenates were stored at −20 °C until the extraction process.

Fruit homogenates obtained from at least 1 kg of fruit were mixed with ethanol containing 1% formic acid (under the conditions listed in Table 1) to obtain fruit extracts. These conditions were selected based on previously reported methods optimized for the extraction of phenolic compounds from the same fruits or fruits containing the same main families of these compounds. The extraction method used for cherry, plum, and strawberry was the one optimized by Iglesias-Carres et al.;^23^, for persimmon kaki and orange was the one optimized by Iglesias-Carres et al. 2019;^24^ and for grapes and pomegranates was the one optimized by Iglesias-Carres et al.^25^ The fruit solutions were vigorously stirred in a Max400 orbital shaker (Thermo Fisher Scientific, Madrid, Spain) at 250 rpm at the times and temperatures listed in Table 1. After extraction, the mixtures were cooled to room temperature and centrifuged (7871*g*, 4 °C, 10 min). Ethanol was removed from the supernatants using a Hei-VAP rotary evaporator (Heidolph, Schwabach, Germany) at 40 °C, and the obtained samples were immediately frozen and lyophilized. Each dried fruit extract was obtained by mixing the powders obtained from at least two different fruit extractions to obtain a unique batch for each fruit extract. Dried extracts were stored at −20 °C in several containers containing the amount of extracts daily needed for animal experiments or other analyses. The yield of the extraction process was calculated by dividing the weight of each dried extract by the weight of the dried fruit and was expressed as a percentage (g wt/100 g wt) (Table S1). Yields ranged between 29 and 87%, with the highest values for grape, cherry, and orange extracts, and the lowest for apricot and plum extracts.

**Table 1:** Extraction Conditions Used for Each Fruit

fruit	solvent/solute ratio (mL/g)	ethanol (%, v/v)	time (min)	temperature (°C)
cherry	20	72	20	55
plum	20	72	30	55
apricot	20	72	30	38
strawberry	40	72	30	55
persimmon kaki	20	72	30	38
grape	80	65	100	72
orange	30	70	40	55
pomegranate	80	65	100	72

### 2.3 Proximate and Phenolic Composition of Fruits and Extracts

Ash, protein, fat, and soluble and insoluble dietary fiber contents in the dried fruit homogenates and dried fruit extracts were determined according to the official methods of analysis of the Association of Official Analytical Chemists (AOAC).^26^ The ash content was calculated as the inorganic residue remaining after the water and organic matter were removed by heating (550 °C for 24 h). Protein content was quantified using the Kjeldahl method with a conversion factor of 6.25.^26,27^ Soluble and insoluble fiber contents were determined by treating the sample with heat-stable α-amylase, protease from *B. licheniformis*, and amyloglucosidase from *A. niger*, and subsequent precipitation of fiber with ethanol according to the AOAC protocol.^26^ The total lipid content of the samples was determined by the Folch method.^28^ For that, samples were prepared according to the method described by Hewavitharana et al.^29^ as follows: lipids were extracted from the samples (5%, w/v) using chloroform/methanol (2:1, v/v), and the lower phase was recovered after the addition of a saline solution. Total (poly)phenol, anthocyanin, and flavanol contents were analyzed following the methodology described by Iglesias-Carres et al.^23^ Dried fruit homogenates and fruit extracts (10 mg) were suspended in 1 mL of a mixture of 1.48% HCl/methanol (60:40, v/v) to determine the total (poly)phenol content, and in Milli-Q water for the other two determinations. HCl was added to eliminate protein interference.^30^ The mixture was vigorously stirred and centrifuged for 2 min at 15,500*g*. The results are expressed as mean ± standard deviation (SD) and expressed as mg of gallic acid equivalents (GAE)/g of dried product, catechin equivalents (CatE)/g of dried product, and cyanidin-3-*O*-rutinoside equivalents (Cy3RE)/g of dried product for the total (poly)phenol, flavanol, and anthocyanin contents, respectively. All analyses were performed in triplicate. The proximate phenolic compositions of the fruit homogenates are shown in Table S2.

### 2.4 Identification and Quantification of Phenolic Compounds in Fruit Extracts by HPLC-Diode Array Detection-Electrospray Ionization-Tandem mass spectrometry (DAD-ESI-MS/MS)

Lyophilized cherry, plum, and strawberry extracts were dissolved (0.5 mg/mL) in methanol/water/acetic acid (70:29:1, v/v/v); lyophilized apricot, persimmon kaki, grape, and pomegranate extracts were dissolved in methanol/dimethyl sulfoxide/water (40:40:20, v/v/v) and 0.1% HCl; and lyophilized orange extract was dissolved in methanol/water (80:20, v/v) and 0.1% formic acid. All mixtures were vigorously stirred for 30 min, centrifuged (4 °C, 15 min, 20,000*g*), and filtered through a 0.22 μm poly(vinylidene dichloride) (PVDF) device (Agilent Technologies, Santa Clara, CA) prior to their injection (20 μL) into a Poroshell 120 EC-C18 analytical column (100 × 3.0 mm^2^, 2.7 μm) from Agilent Technologies. The HPLC-grade solvents used as the mobile phase were water/formic acid (95:1, v/v) (phase A) and acetonitrile (phase B), and they were mixed according to the following gradient: 5–60% B in 37 min, 60–98% B in 3 min, and maintained for 2 min before returning to the initial conditions. The flow rate was set at 1 mL/min. The column was integrated into an Agilent 1100 series HPLC-DAD-ESI-MS/MS system (Agilent Technologies) coupled with a DAD detector and mass spectrometer equipped with an ion trap and electrospray ionization (ESI) interphase. The ESI temperature and capillary voltage were 350 °C and 3500 V, respectively. Helium was used as collision gas (50% collision energy). The mass interval for precursor ions (MS) and subsequent fragments (MS/MS) was 100–1000 *m*/*z*, and data were acquired using the negative ionization mode. *p*-Coumaric acid, quercetin, ellagic acid, pelargonidin, catechin, castalagin, apigenin, hesperidin, and naringenin were used as standards for quantification, and the following absorbance spectra were used: 280 nm for catechin and castalagin, 320 nm for *p*-coumaric, 340 nm for apigenin, hesperidin, and naringenin, 360 nm for quercetin and ellagic acid, and 520 nm for pelargonidin.

### 2.5 Determination of Antioxidant Activity

The antioxidant activity of the extracts was measured using 2,2-diphenyl-1-picrylhydrazyl (DPPH^•^) scavenging capacity, as described by Mau et al.^30^ Different sample concentrations (0.02–1 mg/mL) were tested against DPPH^•^ (76 μM), and the absorbance at 517 nm was recorded after of 15 min incubation at room temperature. IC_50_ was calculated using the linear correlation obtained with increasing sample concentrations, and is referred to as the Trolox equivalent (TE). Results were expressed as Trolox equivalent antioxidant capacity (TEAC).

### 2.6 Experimental Procedure in Rats

A total of 144 male F344 rats, 8 weeks of age, with body weight (BW) approximately 250 g, were purchased from Charles River Laboratories (L’Arblese, Cedex, France) and fed a standard chow diet (AO4, Panlab, Barcelona, Spain) and tap water *ad libitum* throughout the experiment. Despite other laboratory rats’ strain do not respond to daylight length, F334 is a sensitive strain.^31,32^

**Figure 1:**
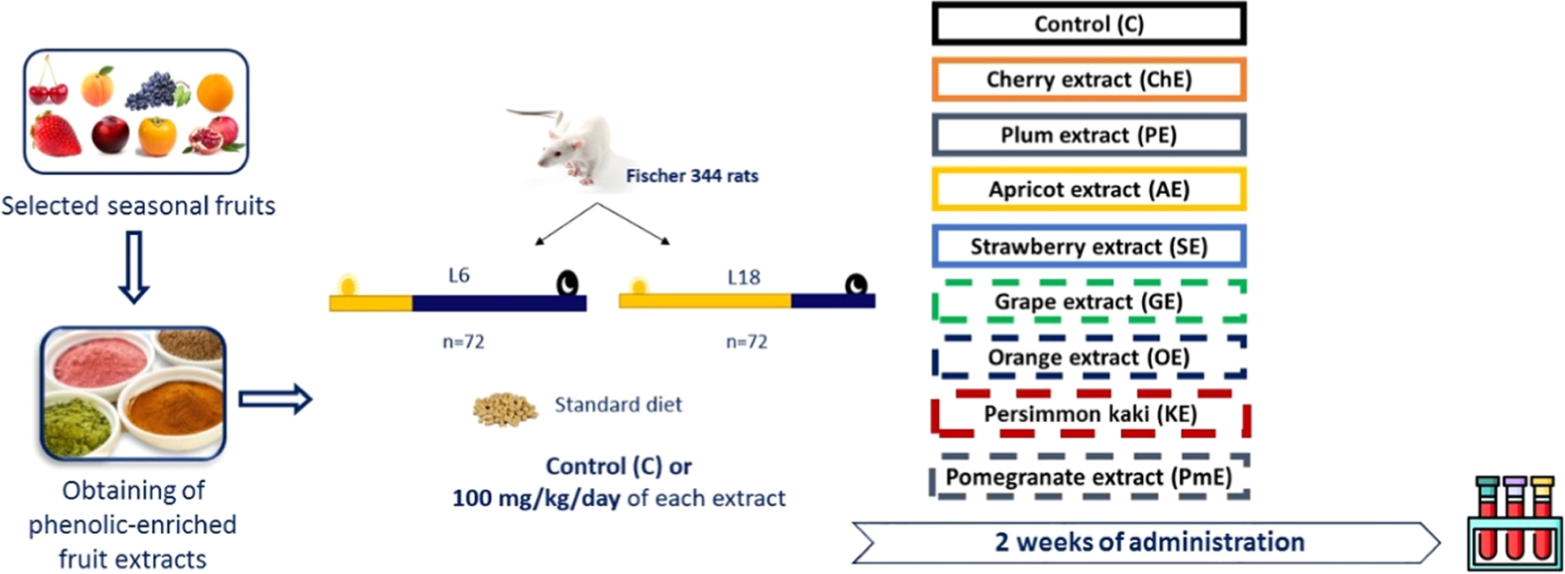
Experimental design used in this study.

Animals were adapted for 1 week and randomly housed into two groups (*n* = 72 per group) and subjected to L6 or L18 photoperiods, simulating winter and summer seasons, respectively, in which lights were turned on at 7:00 a.m. After 4 weeks of adaptation to both photoperiods, rats were administered 100 mg/kg body weight (BW) daily of each extract for 2 weeks (*n* = 8 per group). A group supplemented with 1 mL of tap water was used as the control (C) for each photoperiod. Supplementation was performed by voluntarily liking a syringe between 8:00 and 9:00 a.m. The batch used for each fruit extract was the same for animals subjected to the L6 and L18 photoperiods. Rats were weighed once a week to monitor animal welfare. After 2 weeks, the rats were deprived of food for 12 h and administered either the extract or water. Blood was collected from a cut tail at 3 h after treatment to measure the blood levels of glucose, TAGs, and cholesterol. For insulin quantification, blood was collected from the saphenous vein 3 h after treatment administration and centrifuged (3000*g* for 15 min at room temperature), and the serum was stored at −80 °C until use. The experimental design is illustrated in Figure 1. All procedures were conducted in accordance with the guidelines established by the Animal Ethics Committee of the Universitat Rovira i Virgili (Tarragona, Spain) and approved by the Generalitat de Catalunya (permission number 11610).

### 2.7 Biochemical Analysis

Blood glucose levels were measured by using a glucometer (Glucomen A. Menarini Diagnostics). The total blood TAG and cholesterol levels were measured using an Accutrend Plus instrument (Roche Diagnostics, Barcelona, Spain). Blood was centrifuged, and serum was used to quantify insulin with an enzyme-linked immunosorbent assay (ELISA) kit rat/mouse Insulin 96-well plate assay (EMD Millipore Corporation, St. Louis, Missouri). The samples were assayed in duplicate, according to the manufacturer’s protocol. The homeostatic model assessment for insulin resistance (HOMA) index was calculated as follows: HOMA = fasting insulin (mU/L) × fasting glucose (mmol/L)/22.5.

### 2.8 Statistical Analysis

Normality and homogeneity were evaluated using the Shapiro–Wilk test and Levene’s test, respectively. Two-way and one-way analyses of variance (ANOVA) were used to determine differences among groups using the post hoc Tukey’s test. Two-way ANOVA is indicated as *P* (photoperiod effect), *E* (extract effect), and *P* × *E* (interaction between photoperiod and extract effects). Asterisk (*) indicates significant differences of a specific treatment (control or extract) between photoperiods (*p* ≤ 0.05). Student’s *t* test was used to estimate significant differences (*p* ≤ 0.05) produced by photoperiod in each treatment. Statistical Product and Service Solutions (SPSS) software (SPSS, Inc., Chicago, IL) was used for the statistical analysis. Outliers were identified using SPSS and removed before statistical analysis.

## 3 Results

### 3.1 Proximate and Phenolic Composition of Fruit and Fruit Extracts

No differences were found in ash and soluble fiber contents between fruit extracts (Table 2), while protein contents were different among them, ranging from 0.18 to 1.03%, with the highest values found in orange extract and the lowest in grape extract. Scarce significant differences were found in fat content, ranging from 2.4% in pomegranate extract to 9.8% in grape extract, as well as for insoluble fiber content, ranging from 1.8% in apricot extract to 9.2% in grape extract. The antioxidant activity of the dried extracts ranged from 10.61 to 174.90 μmol TE/g (Table 3). Pomegranate, grape, and strawberry extracts exhibited the highest antioxidant capacities, whereas, as expected, persimmon kaki extract showed the lowest activity. The total (poly)phenol content of the extracts ranged between 4.40 and 54.79 mg GAE/g of dried extract, with the highest values found in the strawberry, cherry, and orange extracts and the lowest in the persimmon kaki extract (Table 3). The total flavanols in the extracts ranged between 0.09 and 1.92 mg CatE/g of dried extract, with the highest values found in the grape extract > strawberry extract > plum extract. Finally, total anthocyanin content ranged from 0.01 to 0.3 mg Cy3RE/g of dried extract. The highest anthocyanin content was observed in the plum extract, whereas it was not detected in the apricot, persimmon kaki, and pomegranate extracts. All extracts showed a significantly higher total (poly)phenol content per dry mass than the original fruit (Table S2). Specifically, it is worth highlighting that the total (poly)phenol content was >3 times higher in persimmon kaki and orange extracts and more than twice that in strawberry extract, in comparison with the amount found in their respective whole fruits, validating the effectiveness of the extraction treatments for phenolic enrichment. Moreover, the extraction process significantly concentrated the flavanol content in all extracts. Extracts from cherries, plums, and oranges were also enriched in anthocyanins. Regarding the phenolic profile of the fruit extracts, a variety of compounds were found, depending on the fruit used as the raw material (Tables 4 and 5). The anthocyanin family was the most abundant in cherry and grape extracts, flavonols in the apricot extract, flavanols in plum extract, and ellagitanins and ellagic acid in strawberry and pomegranate extracts. Moreover, the most abundant molecules were cyanidin-3-rutinoside (1.13 mg/g dried extract) in the cherry extract, procyanidin dimer (0.98 mg/g dried extract) in the plum extract, quercetin-3-rutinoside (3.75 mg/g dried extract) in the apricot extract, malvidin-3-*O*-monoglucoside (0.45 mg/g dried extract) in the grape extract, didymin (2.98 mg/g dried extract) in the orange extract, ellagic acid rhamnoside and ellagic acid (2.90 and 2.34 mg/g dried extract) in the strawberry extract, and punicalagin and pedunculagin (7.26 and 7.59 mg/g dried extract, respectively) in pomegranate extract. In persimmon kaki extract, different compounds were detected at 360 nm; however, they could not be identified and quantified (Table 5).

**Table 2:** Proximate Composition of the Dried Extracts^A^

fruit extract	protein (%)	fat (%)	insoluble fibers (%)	soluble fibers (%)	ash (%)
cherry	0.85 ± 0.02^b^	4.19 ± 2.59^ab^	2.13 ± 0.96^b^	1.95 ± 0.67^a^	1.02 ± 0.27^a^
plum	0.42 ± 0.01^d^	6.27 ± 0.87^ab^	2.59 ± 0.98^b^	1.22 ± 1.14^a^	0.90 ± 0.31^a^
apricot	0.81 ± 0.01^bc^	8.41 ± 2.92^ab^	1.81 ± 0.04^b^	1.00 ± 1.29^a^	1.85 ± 0.18^a^
strawberry	0.75 ± 0.01^c^	8.47 ± 0.29^ab^	2.24 ± 0.70^b^	1.14 ± 1.19^a^	1.61 ± 0.10^a^
persimmon kaki	0.33 ± 0.01^e^	8.75 ± 3.41^a^	1.69 ± 0.15^b^	1.04 ± 1.29^a^	0.89 ± 0.11^a^
grape	0.18 ± 0.02^f^	9.82 ± 0.25^a^	9.21 ± 3.39^a^	2.49 ± 1.12^a^	1.16 ± 0.01^a^
orange	1.03 ± 0.02^a^	6.45 ± 1.23^ab^	2.17 ± 0.61^b^	1.13 ± 0.74^a^	0.76 ± 0.07^a^
pomegranate	0.48 ± 0.08^d^	2.46 ± 0.30^b^	2.41 ± 0.12^b^	2.95 ± 1.48^a^	1.07 ± 0.02^a^

A Values are expressed as the mean (g/100 g dried extract) ± standard deviation (*n* = 3). Different letters indicate significant differences between groups for each compound (*p* ≤ 0.05; one-way ANOVA, post hoc Tukey’s test).

**Table 3:** Total (Poly)phenol, Flavanol, and Anthocyanin Contents of the Dried Fruit Extracts and Their Antioxidant Activity^A^

fruit extract	total (poly)phenols (mg GAE/g dw)	total flavanols (mg CatE/g dw)	total anthocyanins (mg Cy3RE/g dw)	antioxidant activity (μmol TE/g dw)
cherry	14.50 ± 0.64^d^	0.30 ± 0.00^c^	0.21 ± 0.01^b^	34.79 ± 5.13^e^
plum	19.26 ± 0.02^b^	1.70 ± 0.10^b^	0.29 ± 0.00^a^	56.50 ± 5.47^d^
apricot	9.19 ± 0.02^f^	0.09 ± 0.00^d^	n.d.^d^	26.72 ± 4.14^e^
strawberry	54.79 ± 0.28^a^	1.88 ± 0.03^a^	0.18 ± 0.01^c^	81.78 ± 4.10^c^
persimmon kaki	4.40 ± 0.19^g^	0.10 ± 0.00^d^	n.d.^d^	10.61 ± 3.14^f^
grape	17.44 ± 0.52^c^	1.92 ± 0.12^a^	0.01 ± 0.00^d^	107.62 ± 8.61^b^
orange	12.48 ± 0.39^e^	0.18 ± 0.01^cd^	0.03 ± 0.00^d^	26.11 ± 1.26^e^
pomegranate	12.85 ± 0.41^e^	0.25 ± 0.00^c^	n.d.^d^	174.90 ± 7.29^a^

A Values are expressed as the mean ± standard deviation (*n* = 3). Different letters indicate significant differences between groups in each column (*p* ≤ 0.05; one-way ANOVA, post hoc Tukey’s test). CatE, catechin equivalents; Cy3RE, cyanidin-3-*O*-rutinoside equivalents; GAE, gallic acid equivalents; dw, dried weight; n.d, not detected; TE, Trolox equivalents.

**Table 4:** Phenolic Compounds Composition of Summer-Fruit Extracts^a^

phenolic classification		tentative phenolic identification			fruit extracts			
class	subclass	phenolic compound	[M – H]^−^	RT (min)	ChE (mg/g dw)	PE (mg/g dw)	AE (mg/g dw)	SE (mg/g dw)
flavonoids	anthocyanins	cyanidin-3-glucoside	447	8.3		0.16 ± 0.03		
		cyanidin-3-rutinoside	593	8.7	1.13 ± 0.02	0.11 ± 0.02		
		pelargonidin	431	9.5				0.85 ± 0.02
	dihydrochalcones	3-hydroxyphloretin-2′-*O*-glucoside	451	8.1			0.13 ± 0.01	
	flavanols	procyanidin dimer B1	577	6.3		0.98 ± 0.22		
		procyanidin dimer	577	8		1.16 ± 0.26		
		procyanidin dimer	577	10.8		0.98 ± 0.22		
		procyanidin dimer B2	577	10				3.13 ± 0.21
	flavonols	isorhamnetin-3-*O-*glucoside	477	7.3	0.02 ± 0.00			
		kaempferol-3-*O*-glucuronide	461	8.7			0.78 ± 0.12	0.08 ± 0.00
		kaempferol-3-*O*-rutinoside	593	13.5	0.02 ± 0.00			
		quercetin-3-glucuronide	477	12.2				0.04 ± 0.00
		quercetin-3-glucoside	463	12		0.06 ± 0.01		
		quercetin pentosyle-pentoside	565	13		0.02 ± 0.00		
		quercetin-3-xyloside	433	13.3		0.04 ± 0.01		
		quercetin-3-rhamnoside	447	13.7		0.01 ± 0.00		
		quercetin-3-rutinoside	609	11.6		0.04 ± 0.01	3.75 ± 0.23	
		quercetin-3-rutinoside	609	11.7	0.04 ± 0.01			
		ni	403	10.9			1.32 ± 0.10	
		ni	433	12.6		0.01 ± 0.00		
		ni	463	12.6			0.83 ± 0.05	
		ni	505	14.8			0.72 ± 0.10	
		ni	523	17.9			0.72 ± 0.04	
phenolic acids	hydroxybenzoic acids	ellagic acid	301	11.8				2.34 ± 0.08
		ellagic acid pentoside	433	0.8				1.22 ± 0.02
		ellagic acid rhamnoside	447	11.2				2.90 ± 0.03
		ellagic acid rhamnoside	447	11.5				2.90 ± 0.03
		pedunculagin	783	4				0.55 ± 0.04
		pedunculagin	783	6				0.67 ± 0.01
	hydroxycinnamic acids	*cis*-3-*O*-caffeoylquinic acid	353	5.3	0.12 ± 0.00			
		*cis*-3-*O*-caffeoylquinic acid	353	6.1			0.23 ± 0.02	
		*cis*-3-*O*-caffeoylquinic acid	353	8			0.30 ± 0.03	
		*cis*-3-*O*-coumaroylquinic acid	337	6.7	0.02 ± 0.00			
		methyl-3-*O*-caffeoylquinate	367	7.7	0.03 ± 0.00			
		*p*-coumaric acid hexoside	325	7.4		0.02 ± 0.00		0.65 ± 0.00
		chlorogenic/neochlorogenic acid	353	5.5		0.17 ± 0.07		
		ni	611	8.2	0.08 ± 0.00			

a Values are expressed as mean (ppm) ± standard deviation. Anthocyanins, dihydrochalcones, flavanols, flavanones, flavones, flavonols, hydroxybenzoic acids, and hydroxycinnamic acids were quantified using pelargonidin, *p*-coumaric acid, catechin, naringenin, apigenin, quercetin, castalagin, and *p*-coumaric acid standards, respectively. AE, apricot extract; ChE, cherry extract; dw, dried weight; ni, non-identified; PE plum extract; RT, retention time; SE, strawberry extract.

**Table 5:** Phenolic Compounds Composition of Winter-Fruit Extracts^a^

phenolic classification		tentative phenolic identification			fruit extracts			
class	subclass	phenolic compound	[M – H]^−^	RT (min)	GE (mg/g dw)	OE (mg/g dw)	PmE (mg/g dw)	KE (mg/g dw)
flavonoids	anthocyanins	cyanidin-3-glucoside	447	8.3			0.10 ± 0.01	
		malvidin-3-acetylmonoglucoside	533	13.3	0.27 ± 0.04			
		malvidin-3-*O*-*p*-coumarylmonoglucoside	637	16.7	0.17 ± 0.06			
		malvidin-3-*O*-monoglucoside	491	9.8	0.45 ± 0.05			
	flavanols	catechin	289	6.9	<0.01			
		epicatechin	289	8.7	<0.01			
		procyanidin dimer B1	577	6.3	0.08 ± 0.01			
		procyanidin dimer	577	8	0.15 ± 0.10			
	flavanones	didymin	593	9.4		2.98 ± 0.01		
		hesperidin	609	8.6		0.21 ± 0.02		
		hesperidin	609	14.6		1.09 ± 0.08		
		naringenin-7-rutinoside-4′-*O*-glucoside	741	9.9		0.85 ± 0.01		
		naringenin-7-rutinoside	579	13.2		1.71 ± 0.03		
		ni	587	10.3		0.35 ± 0.01		
	flavones	apigenin-7-*O*-(6″-malonyl-apiosyl-glucoside)	649	12.3		0.32 ± 0.00		
	flavonols	laricitrin-3-*O*-glucuronide	507	14.1	0.02 ± 0.00			
		laricitrin-3-*O*-hexoside	493	12.1	0.01 ± 0.00			
		myricetin-3-*O*-galactoside	479	10.3	0.02 ± 0.00			
		quercetin-3-*O*-galactoside	463	7.5	0.01 ± 0.00			
		quercetin-3-*O*-glucoside	463	11.9	0.03 ± 0.00			
		quercetin-3-*O*-(6″-malonyl-glucoside)	549	19.6	0.01 ± 0.00			
		quercetin-3-*O*-(6″malonyl-glucoside)-7-*O*-glucoside	711	17.6		0.01 ± 0.00		
		ni	509	10.5	0.02 ± 0.00			
		ni	551	11.7	0.02 ± 0.00			
		ni	655	14.8	0.02 ± 0.00			
		ni	671	18.2	0.01 ± 0.00			
		ni	323	10.3				nq
		ni	595	10.7				nq
		ni	479	13.2				nq
		ni	631	13.7				nq
		ni	557	14.4				nq
		ni	463	14.7				nq
		ni	463	15				nq
		ni	615	15.2				nq
		ni	615	15.5				nq
		ni	447	16.1				nq
		ni	447	16.8				nq
		ni	599	17.4				nq
phenolic acids	hydroxybenzoic acids	dimethyl ellagic acid hexoside	491	12.0			0.24 ± 0.07	
		ellagic acid	301	11.8			0.13 ± 0.03	
		ellagic acid-deoxyhexoside	447	11.3			0.70 ± 0.13	
		ellagic acid hexoside	463	9.3			0.90 ± 0.00	
		ellagitanins II	799	7.2			2.49 ± 0.20	
		galloyl-HHDP-hexoside	633	8.6			1.98 ± 0.20	
		pedunculagin	783	6			7.59 ± 0.91	
		digalloyl-HHDP-hexoside	783	8.1			3.25 ± 0.33	
		punicalagin	541	5			7.26 ± 0.86	
	hydroxycinnamic acids	5–5′-dehydrodiferulic acid	385	6.4		0.06 ± 0.00		
		5–8′-dehydrodiferulic acid	385	7.5		0.18 ± 0.00		
		Feruloyl glucose	355	8.7		0.11 ± 0.00		

a Values are expressed as mean (ppm) ± standard deviation. Anthocyanins, dihydrochalcones, flavanols, flavanones, flavones, flavonols, hydroxybenzoic acids, and hydroxycinnamic acids were quantified using pelargonidin, *p*-coumaric acid, catechin, naringenin, apigenin, quercetin, castalagin, and *p*-coumaric acid standards, respectively. dw, dried weight; GE, grape extract; ni, non-identified; nq, non-quantifiable; OE, orange extract; PmE, pomegranate extract; KE, persimmon kaki extract; RT, retention time.

### 3.2 Effects of Fruit Extracts on Blood Lipid Profile

**Figure 2:**
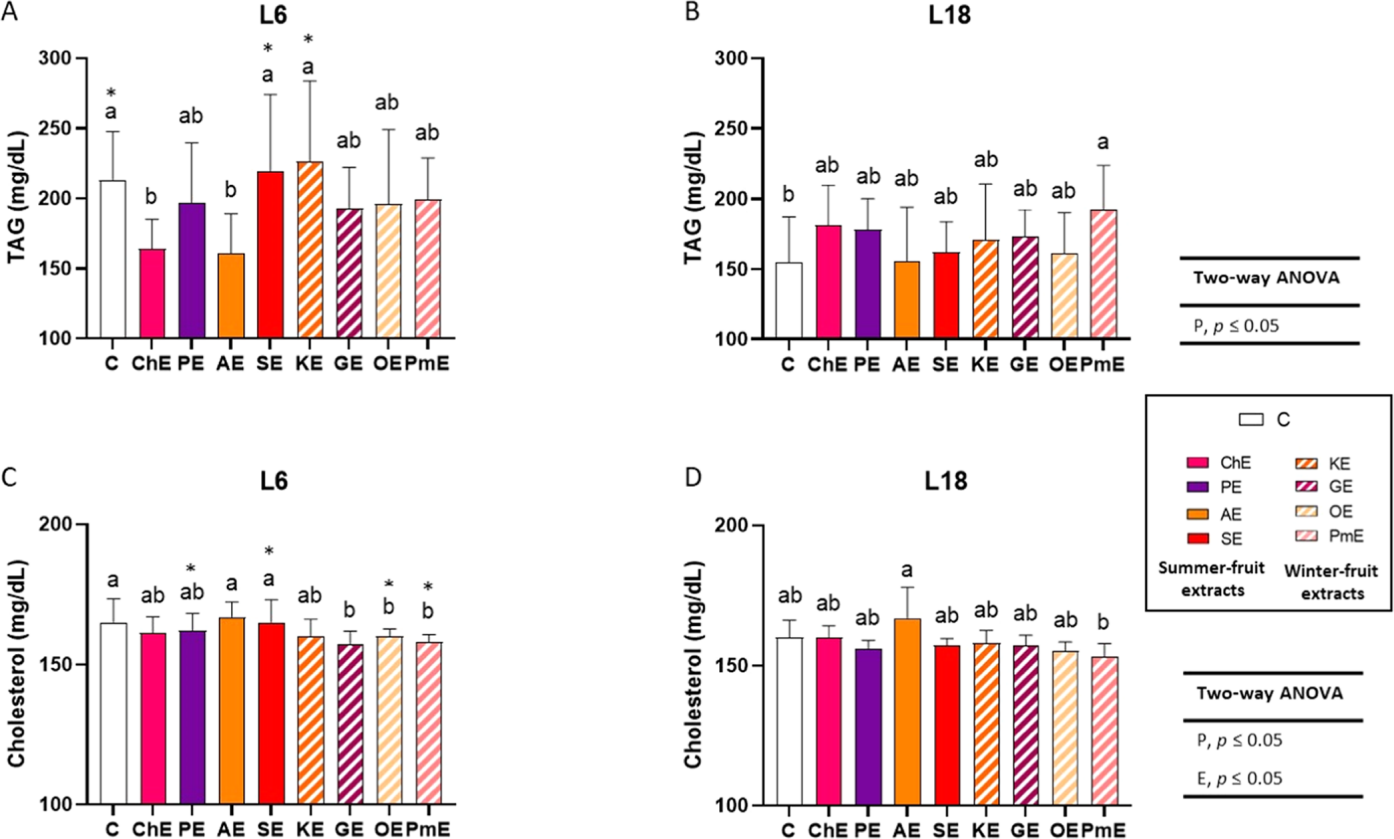
Blood total triacylglyceride (TAG) (A, B) and cholesterol levels (C, D) of Fisher 344 rats exposed to L6 (6 h light) (A, C) and L18 (18 h light) (B, D) photoperiods, which received water (C) or 100 mg/kg body weight of fruit extract (*n* = 8 per group, mean ± standard deviation). Different letters above the bars indicate significant differences among the groups within each photoperiod (*p* ≤ 0.05, one-way ANOVA; post hoc Tukey’s test). Comparisons between photoperiods were carried out using two-way ANOVA (*p* ≤ 0.05, post hoc Tukey-HSD test) and indicated as *P* (photoperiod effect); *E* (fruit extract effect); *P* × *E* (interaction between photoperiod and extract effect). Asterisks indicate significant differences produced by a specific treatment between photoperiods (*p* ≤ 0.05, Student’s *t* test). Fruit extracts: cherry (ChE), plum (PE), apricot (AE), strawberry (SE), persimmon kaki (KE), grape (GE), orange (OE), and pomegranate (PmE) extracts.

Exposure to different photoperiods had a significant effect on blood TAG levels, with higher levels found in animals under the L6 photoperiod than under the L18 photoperiod (two-way ANOVA, *P*, *p* ≤ 0.05) (Figure 2A,B). Moreover, L6-exposed animals showed higher TAG levels only when the control groups were compared (Student’s *t* test, *p* ≤ 0.05). In addition, blood TAG levels were significantly decreased in F344 rats exposed to the L6 photoperiod and administered cherry and apricot extracts compared to those in the L6-C group (Figure 2A). No differences in this parameter were observed for other extracts. In the L18 photoperiod, none of the extracts reduced TAG levels in the treated animals compared with the L18-C group. In contrast, pomegranate extract significantly increased TAG levels in L18-exposed rats (Figure 2B), with no significant difference between this group and L6-exposed rats administered either water or pomegranate extract (Figure 2A). In addition, a photoperiod-dependent differential effect was observed in animals consuming strawberry and persimmon kaki extracts, with higher TAG values observed during the L6 photoperiod than during the L18 photoperiod (Student’s *t* test, *p* ≤ 0.05) (Figure 2A,B).

The effects of photoperiod and extract consumption on blood cholesterol levels were observed when the L6 and L18 groups were compared (two-way ANOVA, *P* and *E*, *p* ≤ 0.05). A photoperiod-dependent effect was observed in L6- and L18-exposed rats after the intake of plum, strawberry, orange, and pomegranate extracts, with lower levels in the L18 photoperiod (Student’s *t* test, *p* ≤ 0.05) (Figure 2C,D). Cholesterol levels decreased when rats consumed three of the four winter-fruit extracts (grape, orange, and pomegranate extracts) compared to the C group during the L6 photoperiod (one-way ANOVA, *p* ≤ 0.05) (Figure 2C). However, none of the extracts decreased cholesterol levels in animals exposed to the L18 photoperiod compared to those in the L18-C group (one-way ANOVA, *p* > 0.05) (Figure 2D).

### 3.3 Effects of Fruit Extracts on Blood Glucose and Insulin Levels

**Figure 3:**
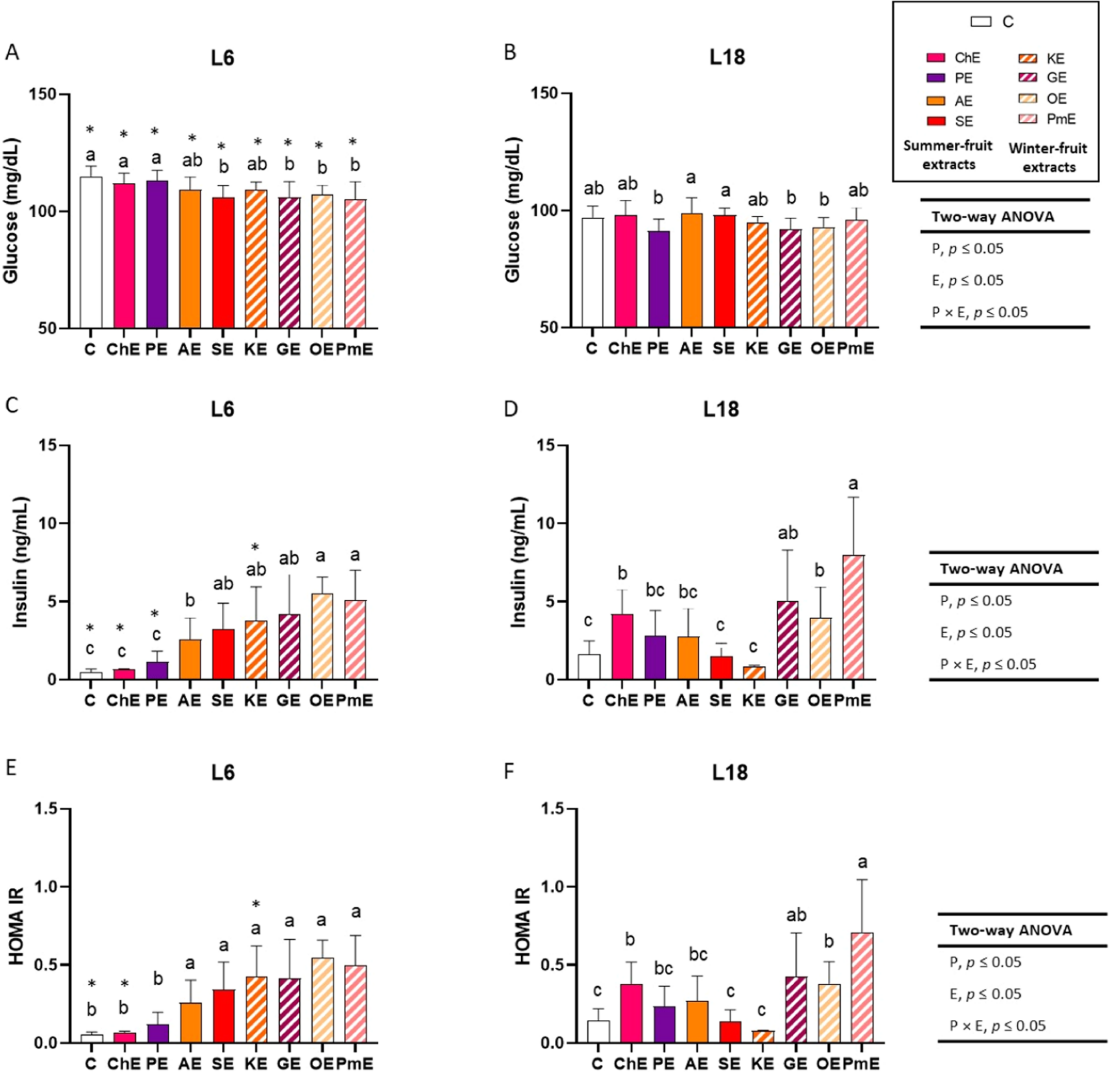
Blood glucose (A, B) and insulin levels (C, D), and HOMA index (E, F) of Fisher 344 rats exposed to L6 (6 h light) (A, C, E) and L18 (18 h light) (B, D, F) photoperiods, which received water (C) or 100 mg/kg body weight of fruit extract (*n* = 8 per group, mean ± standard deviation). Different letters above the bars indicate significant differences among the groups within each photoperiod (*p* ≤ 0.05, one-way ANOVA; post hoc Tukey’s test). Comparisons between photoperiods were carried out using two-way ANOVA (*p* ≤ 0.05, post hoc Tukey-HSD test) and indicated as *P* (photoperiod effect); *E* (fruit extract effect); *P* × *E* (interaction between photoperiod and extract effect). Asterisks indicate significant differences produced by a specific treatment between photoperiods (*p* ≤ 0.05, Student’s *t* test). Fruit extracts: cherry (ChE), plum (PE), apricot (AE), strawberry (SE), persimmon kaki (KE), grape (GE), orange (OE), and pomegranate (PmE) extracts.

A significant effect of photoperiod was observed on glucose levels in the control L6- and L18-exposed animals (Figure 3A,B). Fasting blood glucose levels were affected by photoperiod exposure and the type of extract consumed, and an interaction between both factors was found (two-way ANOVA *P*, *E*, *P* × *E*, *p* ≤ 0.05). Most winter-fruit extracts (grape, orange, and pomegranate extracts), except for the persimmon kaki extract, reduced blood glucose levels in animals exposed to the L6 photoperiod compared to the C group (one-way ANOVA, *p* ≤ 0.05). However, only the summer-fruit extract obtained from strawberries reduced glucose levels in the L6 photoperiod (Figure 3A). No effects on fasting blood glucose levels were observed in L18-exposed animals consuming the different fruit extracts compared with those in the L18-C group (one-way ANOVA, *p* > 0.05) (Figure 3B). Moreover, L6-exposed animals that consumed all extracts showed higher blood glucose levels than did L18-exposed animals that consumed the same extracts (Student’s *t* test, *p* ≤ 0.05) (Figure 3A,B). Insulin levels were also affected by photoperiod exposure and the type of extract consumed, and an interaction between both factors was found (two-way ANOVA, *P*, *E*, *P* × p*E*, *p* ≤ 0.05). Photoperiod effects were observed in control animals and rats consuming cherry, plum, and persimmon extracts, with higher values found in L18-exposed animals than in L6-exposed animals, except for persimmon extract, which showed the opposite effect (Student’s *t* test, *p* ≤ 0.05) (Figure 3C,D). Administration of apricot, strawberry, persimmon, grape, orange, and pomegranate extracts to L6-exposed rats increased blood insulin levels compared to those in the L6-C group (one-way ANOVA, *p* ≤ 0.05) (Figure 3C). In L18-exposed animals, cherry, grape, orange, and pomegranate extracts increased insulin levels compared to those in the L18-C group (one-way ANOVA, *p* ≤ 0.05) (Figure 3D). Overall, the HOMA index showed a pattern similar to that of insulin levels (two-way ANOVA *P*, *E*, *P* × *E*, *p* ≤ 0.05) (Figure 3E,F). Nevertheless, in this case, the photoperiod effect was only observed in control animals and in rats administered cherry and persimmon extracts (Student’s *t* test, *p* ≤ 0.05) (Figure 3E,F). As observed for insulin levels, rats that consumed pomegranate extract showed the highest HOMA value, which was observed during the L18 photoperiod.

## 4 Discussion

According to the Food and Agriculture Organization of the United States, 14% of the food was lost after harvest in 2019.^33^ The greatest losses are from fruits and vegetables,^3^ and strategies to avoid these losses are required. Fresh fruits are a useful source of bioactive compounds that can be extracted,^34^ and phenolic compounds are one of the main compounds.^35^ Phenolic compounds have been shown to exert a wide range of beneficial effects.^36^ Thus, fruit extracts rich in bioactive compounds could be used as functional ingredients for the prevention of several noncommunicable diseases.^37^ Moreover, the elaboration of fruit extracts would allow the fruit-derived phenolic compounds to be consumed in any season of the year, even if the fruit were not available at that time. However, recent animal studies have shown that the health effects associated with the consumption of different phenolic-rich fruits and a grape seed proanthocyanidin extract (GSPE) can differ depending on the photoperiod in which they are consumed.^19,20,38,39^ Advances in the knowledge about the interaction between photoperiod and the functionality of bioactive compounds might be of interest to the scientific community as well as the nutraceutical and food industry to design functional ingredients that could be specific for each season, advancing the concept of personalized nutrition, which is always a concept of high interest in the industrial sector. Considering these facts, the purpose of this study was to obtain phenolic-enriched extracts from seasonal fruits that could modulate blood biochemical markers in F344 rats, and to evaluate whether these effects could be affected by the photoperiod of consumption. The findings in F344 rats show promising evidence regarding the use of some of the developed fruit extracts as potential functional ingredients for the management of blood lipid and glucose levels. Moreover, the obtained results showed the importance of photoperiod consumption in the effectiveness of phenolic-enriched fruit extracts, which was rarely considered in the study of the biological properties of phenolic extracts and other functional ingredients.

Initially, eight ethanol-base extracts were obtained from eight selected seasonal fruits. Phenolic compounds are soluble in organic solvents, and extraction with ethanol, methanol, acetone, or a combination of these with water is the most used method.^40^ This extraction process breaks down plant cell walls, and polymeric complexes are freed into low-molecular-weight compounds that show higher bioavailability.^41^ Ethanol was chosen for the generation of fruit extracts, as it is generally recognized as a safe (GRAS) substance that can be used by the food industry.^42^ In addition, ethanol extraction is widely used to prepare functional extracts.^43^ The present results showed that ethanol-based extractions at low temperatures and in short times allowed us to obtain phenol-enriched extracts from all evaluated fruits. This enrichment was evidenced by the increase in total (poly)phenol levels when extracts were compared to the original fruits, and, although mass extraction yields were significantly high in some cases (Table S1), and lower enrichment might be expected. The extraction conditions have been demonstrated to be effective in releasing additional phenolic compounds from plant/fruit matrices *via* the degradation of cell wall components (particularly adding formic acid). Moreover, a low pH can also lead to depolymerization events that may generate higher total phenolic counts after the extraction process.^23,44^ Differences in fruit species, pre- and postharvest conditions, and fruit parts used to obtain the extract and extraction conditions can explain the variations in phenolic content between the extracts.^43,45^ Interestingly, the extraction conditions used in this study are easily replicable on an industrial scale, which could facilitate the potential transfer of this methodology to the food industry to produce phenol-enriched fruit extracts for use as functional ingredients. Similar values of total (poly)phenol content of fruit extracts have been reported for strawberry extracts (1.3–2.7 mg GAE/g dried extract) and pomegranate juice and extracts obtained from pomegranate seeds and peels (12.0–276 mg GAE/g dried extract).^46^ However, higher values of total phenolics have been reported for other fruit extracts, but they were obtained using a higher extraction time than in the present study (2 days vs 20–100 min, respectively).^43^ Spigno et al. also reported an increase in the total polyphenol content of extracts from red grapes when the extraction time was increased from 1 to 20 h at both 45 and 60 °C, with the highest values at 60 °C.^45^

Moreover, the phenolic profiles of the different fruit extracts were analyzed. As expected, the phenolic profile of the extracts varied depending on the fruit species used to prepare the extract. In most cases, this phenolic profile did not coincide with that of the other reported extracts obtained from the same fruit. As mentioned above, different factors, such as fruit species and variety, soil type, environmental conditions, storage conditions, ripeness degree, and extraction conditions can affect this profile.^18,47^ For instance, flavanone was the most relevant phenolic family in the orange extract, as in other citrus juices, ranging from 81 and 97% of the total phenolic composition.^48^ Specifically, didymin was the major phenolic compound in the extract. This compound was also identified in other extracts obtained from sweet orange pulp under the same extraction conditions as in the current study. However, unlike the obtained extract, these other reported ones also contained high levels of kaempferol-3-*O*-rutinoside.^24^ A study of the phenolic composition of 27 sweet orange juices (*C. sinensis*) showed that this kaempferol derivative can be present in different concentrations, even absent, in orange juices, depending on the orange variety and campaign.^48^ Moreover, anthocyanin cyanidin-3-rutinoside was the main phenolic compound found in the cherry extract; however, rutin was the most representative compound found in an ethanolic extract obtained from Royal dawn sweet cherry.^23^ In contrast to this reported study, other methanolic extracts obtained from different sweet cherry varieties contained cyanidin-3-rutinoside and epicatechin as the main phenolic compounds, and rutin was not identified in these extracts.^49^ Regarding grape, strawberry, and pomegranate extracts, the most relevant compounds found in these extracts coincided with other reported extracts, although their concentrations differed. In this regard, a higher concentration of procyanidin B2 was found in the strawberry extract than that previously reported for other strawberry extracts,^50^ whereas lower levels of malvidin-3-*O*-glucoside were found in the grape extract than the ones found in other grape extract obtained under the same extraction conditions.^25^ Similarly, although ellagitannin was the most relevant family found in the pomegranate extract, a lower concentration of anthocyanins was found in this extract with respect to the ones observed in other pomegranate extracts.^51^

One of the most important properties of phenolic compounds is their antioxidant capacity.^52^ Fresh fruits, such as strawberries,^53^ grapes,^54^ plums,^55^ and pomegranate,^56^ have shown high antioxidant activity, which is correlated with the abundance of phenolic compounds. Thus, this bioactivity was evaluated in the extracts through their DPPH^•^ scavenging capacity *in vitro*. All eight extracts showed DPPH^•^ scavenging capacity, with the pomegranate extract being the most active. Although this extract did not show the highest (poly)phenolic content (12.85 mg GAE/g), its richness in specific molecules such as hydrobenzoic acids, *e.g.*, pedunculagin and punicalagin, might be crucial to exert the reported ability as free radicals scavenger. Other studies have also reported high antioxidant activity, as measured by the β-carotene bleaching test of aqueous ethanolic extracts (80%, 25 °C, 48 h) of peel, seed, and juice from different varieties of pomegranate.^57^ In addition to pomegranate, high antioxidant capacity has been reported for other fruit extracts, such as an aqueous ethanol cherry extract (70%, room temperature, 2 h), measured as nitric oxide scavenging activity.^58^

In addition to their antioxidant properties, phenolic compounds play a key role in the regulation of several metabolic processes, including protein regulation and gene expression, and their effects can differ depending on the photoperiod of consumption.^18^ In the present study, two summer-fruit extracts (cherry and apricot extracts) decreased TAG levels compared to control-L6-exposed rats and the winter-fruit extracts (grape, orange, and pomegranate extracts) reduced total cholesterol levels under a short photoperiod, suggesting that this effect can only be observed when consumed in winter-like conditions. To the best of our knowledge, this is the first study to report the photoperiod-dependent effects of fruit extracts. The beneficial effects of these extracts might be associated with their phenolic profile, considering that these compounds are enriched in the extract with respect to the whole fruit and are the main compounds associated with the health effects of fruits and vegetables. However, it should not be ruled out that other fruit compounds, such as fructose, may also be involved in the effects of the fruit extracts. Cyanidin-3-*O*-rutinoside could be involved in the effects of the cherry extract given this compound, the majority of which, can inhibit several enzymes *in vitro*, such as pancreatic cholesterol esterase and lipase, and reduce cholesterol uptake in Caco-2 cells.^59^ Similarly, rutin (quercetin-3-*O*-rutinoside), the main phenolic compound in the apricot extract, also inhibited pancreatic lipase *in vitro* and reduced the lipid content and adipogenesis of 3T3-L1 cells.^60^ Total cholesterol levels have also been shown to be modulated by ellagitanins present in the pomegranate extract when administered to rats fed a high fructose diet.^61^ In addition, the obtained orange extracts showed relevant levels of flavanones, such as didymin, an antioxidant compound with beneficial effects on cardiovascular health, for instance, by inhibiting lipid peroxidation.^62^ Finally, anthocyanins could be involved in the effects of the grape extract, given that there is a correlation between the clinical supplementation of anthocyanins and the reduction of low-density lipoprotein cholesterol and cardiovascular risk.^63^ Previous studies have reported that the consumption of some whole fruits by healthy animals improved their blood lipid profile. For instance, rats consuming sun-dried apricots for 120 days switched to a hypotriglyceridaemia state and increased blood total cholesterol and high-density lipoprotein cholesterol (HDL-C) levels in a sex-dependent manner.^64^ Another study reported that sweet cherry consumption for 7 weeks also improved the plasma lipid profile of F344 animals. However, similar to the present study, their effects depended on the photoperiod of consumption. Specifically, it reduced TAG levels in the L6 photoperiod, while its consumption increased HDL-C and reduced nonesterified fatty acids in the L18 photoperiod.^20^ However, no effects were observed in total cholesterol levels, as did in the present study. It is important to highlight that sweet cherry effects also depended on the composition of the cherries, which could explain the different effects of sweet cherries and the cherry extract generated in the present study on plasma lipid biomarkers and the different effects shown by the different fruit extracts. These differential effects between cherries of the same variety were attributed to their different (poly)phenol signatures.^20^ It is known that different fruit species have different phenolic profile and activities when ingested.^35^ Moreover, interestingly, fruits from the same variety have distinctive phenolic hallmarks depending on the cultivar system, geographical origin, and other factors and display different effects depending on the fruit variety and photoperiod.^16^ Similarly, Gibert-Ramos et al. observed that two oranges of different origins exhibited distinct fat accumulation in F344 rats exposed to L6 and L18 photoperiods, which was attributed to the different concentrations of phenolic compounds in each orange.^65^ Furthermore, no differences were observed in TAG and total cholesterol levels in F344 rats after intake of any of the oranges for 10 weeks compared to the control group.^65^ This is not in agreement with the present results, in which the orange extract reduced total cholesterol levels when consumed under a short photoperiod. These discrepancies could be attributed to differences in the phenolic hallmarks and levels of total (poly)phenols between the whole fruits and extracts. Moreover, in the present study, the (poly)phenol concentrations in cherry and orange extracts were 1.4 and 3.4 times higher, respectively, than those quantified in the corresponding whole fruits. In addition, the bioavailability of phenolic compounds present in the extract can vary with respect to the whole fruit, as they are released from the cellular matrix.^10^

Effects of the fruit extracts on blood glucose levels were also evaluated in F344. Cherry, grape, orange, and pomegranate extracts (3/4 of which were winter-fruit extracts) decreased the blood glucose levels in L6-exposed rats. Phenolic compounds have been shown to control glycemia, which is attributed to the inhibition of salivary and pancreatic enzymes during sugar digestion.^66^ Specifically, some phenolic molecules that are mainly present in the obtained extracts have been reported as potential antidiabetic agents, such as didymin and punicalgin,^67,68^ which are mainly present in orange and pomegranate extracts, respectively. In addition, another study showed that pomegranate and grape extracts could modulate carbohydrate digestive enzyme activity, including *in vitro* inhibition of α-amylase and α-glucosidase.^69^ Photoperiod effects on blood glucose levels were also observed in F344 rats exposed to different photoperiods and consuming the enriched-phenolic grape seed extract GSPE for 9 weeks, although and unlike in the current study, GSPE increased its concentration in the L6 and L18 photoperiods compared to their respective control animals and treated and untreated L12-exposed animals.^21^ In healthy animals, insulin is released by the pancreatic islets after carbohydrate meals to remove glucose from the bloodstream. When elevated levels of insulin are produced owing to the intake of high-glycaemic index foods, an insulin-resistant phenotype can develop.^70^ In the present study, all animal groups were considered insulin-sensitive, with a HOMA index below 1, although most of the extracts tended to increase the HOMA index under fasting conditions (close to 1). Moreover, a differential effect on insulin and the HOMA index was observed after the intake of cherry, plum, and persimmon extracts (plum only for insulin levels) when consumed during different photoperiods. Cherry and plum extracts tended to increase the effect of the photoperiod. However, the pomegranate extract consumed during L6 photoperiod increased the insulin levels, and the HOMA index was higher than that shown by the L18-C group and did not produce any effects when consumed during the L18 photoperiod. These findings are in concordance with those of previous studies, in which serum insulin levels and the HOMA index were higher in F344 rats exposed to a short photoperiod (L6) and consuming sweet cherries for 7 weeks than in control animals, whereas no effects were observed in animals under the L18 photoperiod.^20^ However, in the present study, cherry extracts increased serum insulin levels only during the L18 photoperiod, which could be due to the different profiles and amounts of phenolic compounds between the whole cherry and the cherry extract used in this study.

Evidence shows that the effects of fruit extracts on fasting blood biomarkers are affected by the photoperiod. These differences could be attributed to several factors. For instance, F344 rat metabolism varies depending on the photoperiod, which was evidenced by the significant differences observed between the L6-C and L18-C groups in the current study. Higher TAG and glucose levels and lower insulin and HOMA index values were observed in animals under the L6 photoperiod than under the L18 photoperiod. The existence of metabolic variations due to differential exposure to photoperiods was previously observed in this animal model.^71^ Moreover, it was reported that the bioavailability of fruit (poly)phenols can be modulated in a photoperiod-dependent manner in F344 rats. Iglesias-Carres et al. observed that the bioavailability of grape (poly)phenols was higher in animals exposed to an L6 photoperiod.^72^ This result was in agreement with those reported in other research, in this case targeting GSPE, and additionally demonstrating the crucial role of gut microbiota in phenolic compound bioavailability.^73^ Other fruits, such as tomatoes, also exhibited differential bioavailability of specific (poly)phenols due to the photoperiod of consumption.^74^

In conclusion, several fruit extracts modulated fasting blood lipid, glucose, and insulin levels in a photoperiod-dependent manner, mainly being effective when consumed under a short photoperiod (winter-like). Specifically, the pomegranate, grape, and orange extracts could be useful for their cholesterol-lowering and fasting glucose-lowering properties when consumed under the L6 photoperiod. In addition, plum extract could help maintain fasting glucose levels if consumed during the L18 photoperiod without altering the HOMA index and TAG and cholesterol levels. Finally, cherry and apricot extracts could be useful for decreasing TAG levels only when administered during the L6 photoperiod. In addition, the tested fruit extract dose can be extrapolated to a human dose (1.5 g extract/day, considering a person weighing 70 kg and height of 1.75 m) that easily could be reached through the consumption of functional foods or nutraceuticals based on these fruit extracts.^75^ These results suggest that the season of consumption should be considered in the study of the beneficial effects of phenolic-enriched extracts and in functional ingredient design. Nevertheless, further studies are needed to investigate whether these fruit extracts could be useful in the prevention of hyperlipidemia or hyperglycemia, and to identify the most effective extracts for each photoperiod. Moreover, clinical trials are required to validate the promising results obtained in F344 rats, as these insights must be confirmed in human subjects before establishing dietary recommendations. Additional experiments involving bioaccessibility and bioavailability of the bioactive phenolic compounds considering the matrix, as well as sensory and consumer acceptance analyses, would be crucial for the design of functional foods based on fruit extracts.

## Supporting Information Available

Yield of the extract process and the proximate composition of dried fruits (Tables S1 and S2) (PDF)
