# Supplementary material for: Photoperiod-Dependent Effects on Blood Biochemical Markers of Phenolic-Enriched Fruit Extracts
Source: J Agric Food Chem. 2024 May 29;72(23):13111–24. doi: 10.1021/acs.jafc.4c01698 (PMC11181326; doi:10.1021/acs.jafc.4c01698)
Supplement: Supplementary file 1 — jf4c01698_si_001.pdf [file jf4c01698_si_001.pdf]

SUPPORTING INFORMATION

Photoperiod-dependent effects on blood biochemical markers of phenolic-enriched fruit extracts

Francesca Manocchio[1,2], Diego Morales[1,2,#], Elia Navarro-Masip[1,2], Gerard Aragonès[1,2,3], Cristina Torres-Fuentes[1,2,3], Francisca Isabel Bravo[1,2,3,*], Begoña Muguerza[1,2,3]

[1]Universitat Rovira i Virgili, Departament de Bioquímica i Biotecnologia, Nutrigenomics Research Group, C/ Marcel·lí Domingo s/n, 43007, Tarragona, Spain.

[2]Nutrigenomics Research Group, Institut d'Investigació Sanitària Pere Virgili. C/ Marcel·lí Domingo s/n 43007 Tarragona, Spain

[3]Center of Environmental, Food and Toxicological Technology (TecnATox), University Rovira i Virgili, C/ Marcel·lí Domingo s/n, 43007 Tarragona, Spain

#current affiliation: Departmental Section of Galenic Pharmacy and Food Technology, Veterinary Faculty, Complutense University of Madrid, Avda. Puerta del Hierro s/n, 28040 Madrid, Spain.

***Corresponding author:** Francisca Isabel Bravo (F.I.B), *E-mail: franciscaisabel.bravo@urv.cat, +34977558837

Table S1. Yield of the extract process

**Fruit**	**Yield** (%)
Cherry	83.27 ± 5.09^{ab}
Plum	33.84 ± 0.94^{d}
Apricot	31.26 ± 3.41^{d}
Strawberry	64.66 ± 2.63^{bc}
Persimmon kaki	55.40 ± 2.55^{c}
Grape	86.57 ± 4.05^{a}
Orange	80.07 ± 2.89^{ab}
Pomegranate	73.43 ± 0.77^{b}

The yield of the extraction process was calculated by dividing the weight of each dried extract by that of the used dried fruit. Different letters indicate significant differences between groups ($p \leq 0.05$, one-way ANOVA, post-hoc Tukey's test)

Table S2. Proximate composition of the dried fruits

Fruits	Protein (%)	Fat (%)	Insoluble fiber (%)	Soluble fibers (%)	Ash (%)	Total polyphenols (mg GAE/g dw)	Total flavanols (mg CatE/g dw)	Total anthocyanidins (mg Cy3RE/g dw)
**Cherry**	6.77 ± 0.04 [a]	0.66 ± 0.01 [a]	2.66 ± 0.51 [a]	2.54 ± 1.89	5.48 ± 1.37	10.17 ± 1.03 [a]	0.00 ± 0.00	0.13 ± 0.01 [a]
**Plum**	4.38 ± 0.22 [b]	1.00 ± 0.09 [b]	4.36 ± 0.95 [a]	6.10 ± 1.56	9.03 ± 3.43	12.97 ± 1.86 [a]	0.01 ± 0.00	0.05 ± 0.01 [b]
**Apricot**	6.14 ± 0.07 [a]	0.74 ± 0.00 [ab]	10.22 ± 1.29 [b]	6.77 ± 1.75	9.38 ± 2.36	6.26 ± 0.28 [b]	n.d.	0.01 ± 0.00 [c]
**Strawberry**	8.93 ± 0.09 [c]	0.59 ± 0.00 [a]	15.35 ± 1.88 [b]	6.17 ± 3.22	4.93 ± 0.25	22.92 ± 1.43 [c]	0.01 ± 0.00	0.30 ±0.03 [a]
**Persimmon kaki**	4.20 ± 0.14 [b]	1.11 ± 0.20 [a]	26.81 ± 1.43 [c]	4.17 ± 1.19	2.23± 0.02	1.20 ± 0.03 [d]	0.00 ± 0.00	0.03 ± 0.00 [b]
**Grape**	2.91 ± 0.99 [b]	3.09 ± 0.22 [c]	23.26 ± 3.92 [c]	1.83 ± 0.81	10.05 ±5.49	10.10 ± 1.68 [a]	0.02 ± 0.00	0.08 ± 0.01[b]
**Orange**	6.95 ± 2.47 [a]	2.12 ± 0.04 [c]	19.61 ± 5.41 [c]	15.38 ± 11.23	3.95 ± 1.47	3.62 ± 0.03 [e]	0.00 ± 0.00	0.02 ± 0.00 [c]
**Pomegranate**	7.09 ± 0.89 [a]	2.65 ± 0.35 [c]	13.57 ± 0.18 [b]	4.17 ± 1.29	2.86 ± 1.65	9.79 ± 0.08 [a]	0.00 ± 0.00	0.16 ± 0.01[a]

Values are expressed as the mean ± standard deviation (n=3). Different letters indicate significant differences between groups ($p \leq 0.05$, one-way ANOVA, post-hoc Tukey's test). CatE, catechin equivalents; Cy3RE, cyanidin-3-*O*-rutinoside equivalents; GAE, gallic acid equivalents; dw, dried weight; n.d, not detected. Percentage is represented in g/100 g dw.
